# Interventions to improve early retention of patients in antiretroviral therapy programmes in sub-Saharan Africa: A systematic review

**DOI:** 10.1371/journal.pone.0263663

**Published:** 2022-02-09

**Authors:** Samuel Muhula, John Gachohi, Yeri Kombe, Simon Karanja

**Affiliations:** 1 School of Public Health, Jomo Kenyatta University of Agriculture and Technology, Juja, Kenya; 2 Amref Health Africa, Nairobi, Kenya; 3 Kenya Medical Research Institute, Nairobi, Kenya; University of North Carolina at Chapel Hill, UNITED STATES

## Abstract

**Background:**

Several interventions to improve long term retention (12 months and above) on treatment have been rigorously evaluated in Sub-Saharan Africa (SSA). However, research on interventions to improve retention of patients in the early stages of treatment (6 months) during this era of Universal Test and Treat has only recently emerged. The aim of this study is to systematically map evidence of interventions used to improve early retention of patients in antiretroviral therapy (ART) programmes in SSA.

**Methods:**

We searched PubMed, EMBASE and Cochrane electronic databases to identify studies describing interventions aimed at improving early retention in ART treatment. We applied the methodological frameworks by Arksey and O’Malley (2005) and Levac et al. (2010). We also followed the Preferred Reporting Items for Systematic Reviews and Meta-Analyses (PRISMA) checklist. Interventions were categorized according to key broad areas in the existing literature.

**Results:**

A total of 2,241 articles were identified of which 19 met the inclusion criteria and were eligible for this review, with the majority either being randomized control trials 32% (n = 6) or cohort studies 32% (n = 6). The studies reviewed were conducted in 11 SSA countries. The most common interventions described under key broad areas included: Health system interventions such as Universal Test-and-Treat, integration of ART initiation, HIV Testing and Counselling and Antenatal Care services and reduction of ART drug costs; Patient centered approaches such as fast track ART initiation, Differentiated Drug Delivery models and point of care HIV birth testing; Behavioral interventions and support through lay counselors, mentor mothers, nurse counselors and application of quality improvement interventions and financial incentives. Majority of the studies targeted the HIV positive adults and pregnant women.

**Conclusion:**

With the introduction of Universal Test-and-Treat and same-day initiation of ART, findings suggest that adoption of policies that expand ART uptake with the goal of reducing HIV transmission at the population level, promoting patient centered approaches such as fast track ART initiation, Differentiated Service Delivery models and providing adequate support through Mentor Mothers, lay and nurse counselors may improve early retention in HIV care in SSA. However, these interventions have only been tested in few countries in the region which points to how hard evidence based HIV programming is. Further research investigating the impact of individual and a combination of interventions to improve early retention in HIV care, including for various groups at high risk of attrition, is warranted across SSA countries to fast track the achievement of 95-95-95 Joint United Nations Programme on HIV/AIDS (UNAIDS) targets by 2030.

## 1. Introduction

Retention in care soon after HIV diagnosis is critical in achieving good health outcomes, preventing increased mortality in public health care setting and reduces the risk of viral rebound [1, 2]. Lower retention rates in HIV care serves as a major barrier to optimal HIV care and correlates with worse outcomes and increased transmission of HIV [3]. Despite the continued severity of the HIV epidemic, tremendous achievements have been made in the fight to end the epidemic. However, a significant proportion of persons with HIV still do not have consistent access to antiretroviral therapy (ART), many a time due to decreased engagement in long term clinical care [4]. Eastern and Southern Africa, the region hardest hit by HIV has made strong progress in the expansion of ART although many challenges still exist. In 2018, only 58% of people living with HIV (PLHIV) in the region had viral load suppression with wide disparities ranging from 16% in Mauritius to above 95% in Botswana [5]. Attrition from HIV care, especially in the first months after ART inititaion poses a major challenge in HIV treatment cascade, with an unacceptable number of lost-to-follow-ups (LTFU) after initiating treatment [5, 6]. Major risk factors to LTFU from ART programmes include several sociodemographic (male sex, older age, being single, unemployment, lower educational status), clinical (advanced WHO stage, low weight, worse functional status), patient behaviour (poor adherence, nondisclosure), treatment related and system level factors [7]. A systemic review of a pooled magnitude of HIV patient clinical retention and attrition in Ethiopia found out that major determinants of attrition were being unmarried patient, non-disclosed HIV status, poor drug adherence, poor functional status, being underweight and advanced clinical stage [8]. Knowledge of risk factors should be used to better develop retention interventions in HIV programmes. Attrition from ART treatment is therefore a major driver of poor programme performance and increased morbidity and mortality, affecting the achievement of the UNAIDS 95-95-95 targets by 2030 (95% of people living with HIV know their HIV status, of whom 95% are on antiretroviral treatment and of whom 95% are virally supressed) [9].

Therefore, WHO recommends the development of innovative strategies to help countries fast-track the achievement of the ambitious UNAIDS targets. Early initiation of PLHIV on ART, retaining them on ART and ensuring virological suppression has significant clinical benefits on treatment and can eliminate HIV transmission [10]. Unfortunately, minimal strategies focusing on early retention of patients in HIV treatment programmes have been explored, and high attrition, poor treatment adherence of patients on HIV care and treatment programmes, continue to be a menace [11]. A systematic review of existing evidence on the clinical outcomes of Differentiated Service Delivery (DSD) model for HIV treatment in SSA point to a scarcity of such models in both quantity and quality and suggest that retention in care and viral suppression are roughly equivalent to those in conventional models of care [12].

Several interventions to improve long term retention (12 months and above) on treatment have been rigorously evaluated including policies for Universal Test and Treat (UTT), same-day initiation of ART [13], 6-monthly ART dispensing [14] and community-based adherence clubs for postpartum women [15]. However, research on interventions to improve retention of patients in the early stages of treatment (within 6 months after starting ART) during this era of UTT emerged recently. The aim of this study is to systematically map evidence of interventions used to improve early retention of patients in ART programmes in SSA.

## 2. Methodology

### 2.1. Operational definitions

For this review, “retention” refers to patients known to be alive and receiving highly active ART at six months from the first point of ART initiation (baseline) and measured by whether the patient attended a follow-up appointment within 5 to 7 months timeframe after their first clinic visit.

Hinari is a programme set up by WHO together with major publishers to enable low- and middle- income countries to gain access to one of the world’s largest collections of biomedical and health literature. Up to 21,000 journals, up to 69,000 e-books, up to 115 other information resources are now available to health institutions in more than 125 countries, areas and territories benefiting many thousands of health workers and researchers, and in turn, contributing to improve world health.

### 2.2. Eligibility criteria

Studies/publications of all designs/types were included in this systematic review. Studies were eligible if the target population was HIV positive patients in SSA according to The World Bank country definitions [16, 17], written in English and described the intervention aimed at improving early retention of patients on ART programmes as the primary outcome of interest.

The review excluded study protocols and systematic reviews because they did not have findings yet and did not document findings for a particular intervention but a review of a collection of studies respectively.

A tool for assessing study eligibility was adapted from the Cochrane Data Collection form for intervention reviews for Randomized Control Trial (RCT) and non-RCTs template [18] and included the study characteristics (type of study, participants, types of intervention, type of outcome measures), eligibility criteria (included the inclusion criteria for each characteristic), eligibility criteria met (Yes, No, Unclear), whether to include the study or not (Include, Exclude) and the reason for exclusion.

### 2.3. Information sources

To identify potentially relevant documents, we searched published and unpublished researches documenting interventions targeted at improving early retention of HIV patients on care and treatment in SSA. The studies were retrieved through internet searches from PubMed, EMBASE and Cochrane electronic databases. We also searched for gray literature from Google Scholar and WHO, UNAIDS, the USA and Africa Centers for Disease Control and Prevention (CDC) websites. Articles that met the eligibility criteria and could not be retrieved from the databases were either accessed from Hinari or contacted the authors for the full papers.

### 2.4. Search strategy

We followed the Preferred Reporting Items for Systematic Reviews and Meta-Analyses (PRISMA) checklist and explanation to report the systematic reviews [19]. We used compound search strategy which enabled the combination of columns in a logic grid table using boolean operator AND and combination of key and Medical Subject Headings (MeSH) terms within a given column in the logic grid table using boolean operator OR. Table 1 is the logic grid for the search strategy with key and MeSH terms searched from the online databases. Reference lists of already identified studies were screened to retrieve additional articles. All published articles between 2000 and March 2021 were included in this review. The key terms were standardized across all the databases to produce comparable results.

**Table 1:** The logic grid for search strategy.

Population	Exposure/Intervention	Outcomes	Sub-Saharan countries
**Key terms**	**Key terms**	**Key terms**	**Key terms**
• HIV	• Financial incentive	• Retention	• Angola
• Human immunodeficiency virus	• Cash incentive	• Retention in Care	• Benin
• AIDS	• Monetary	• Retention in Antiretroviral Therapy Programs	• Botswana
• Acquired Immunodeficiency Syndrome	• Non-cash incentive	• Retention in ART	• Burkina Faso
• HIV&AIDS	• None cash incentive	• Retained on treatment	• Burundi
• People Living with HIV	• In-kind incentive	• Retained on HIV treatment	• Cabo Verde
• PLWHIV	• nonmonetary	• Retained on ART	• Cameroon
• PLHIV	• non-monetary		• Central African Republic
• HIV-positive	• Incentive		• Chad
• HIV positive	• Motivation		• Comoros
	• Conditional cash transfer		• Democratic Republic of the Congo
	• CCT		• Djibouti
	• Methods		• Republic of the Congo
	• Intervention		• Congo
	• Financial Support		• Cote d’Ivoire
			• Equatorial Guinea
			• Eritrea
			• Eswatini
			• Ethiopia
			• Gabon
			• Gambia
			• Ghana
			• Guinea
			• Guinea-Bissau
			• Kenya
			• Lesotho
			• Liberia
			• Madagascar
			• Malawi
			• Mali
			• Mauritania
			• Mauritius
			• Mozambique
			• Namibia
			• Niger
			• Nigeria
			• Rwanda
			• Sao Tome and Principe
			• Senegal
			• Seychelles
			• Sierra Leone
			• Somalia
			• South Africa
			• South Sudan
			• Sudan
			• Tanzania
			• Togo
			• Uganda
			• Zambia
			• Zimbabwe
			• sub-Saharan Africa
			• SSA
			• Africa South of the Sahara
**MeSH terms**	**MeSH terms**	**MeSH terms**	**MeSH terms**
• HIV	• Motivation	• Retention in Care	• Benin
• AIDS	• monetary		• Botswana
	• Methods		• Burkina Faso
	• Financial Support		• Burundi
			• Cabo Verde
			• Cameroon
			• Central African Republic
			• Chad
			• Comoros
			• Democratic Republic of the Congo
			• Djibouti
			• Congo
			• Cote d’Ivoire
			• Equatorial Guinea
			• Eritrea
			• Eswatini
			• Ethiopia
			• Gabon
			• Gambia
			• Ghana
			• Guinea
			• Guinea-Bissau
			• Kenya
			• Lesotho
			• Liberia
			• Madagascar
			• Malawi
			• Mali
			• Mauritania
			• Mauritius
			• Mozambique
			• Namibia
			• Niger
			• Nigeria
			• Rwanda
			• Sao Tome and Principe
			• Senegal
			• Seychelles
			• Sierra Leone
			• Somalia
			• South Africa
			• South Sudan
			• Sudan
			• Tanzania
			• Togo
			• Uganda
			• Zambia
			• Zimbabwe
			• Africa South of the Sahara

All references identified through the compound search strategy were imported into EndNote referencing tool and duplicates from the different databases removed. Table 2 shows the search history for PubMed database which is a sample of search history as done in EMBASE and Cochrane databases.

**Table 2:** Search history for PubMed database.

#	Query	Results	Date
#5	((((((((((((((HIV[Title/Abstract]) OR (Human immunodeficiency virus[Title/Abstract])) OR (AIDS[Title/Abstract])) OR (Acquired Immunodeficiency Syndrome[Title/Abstract])) OR (HIV&AIDS[Title/Abstract])) OR (People Living with HIV[Title/Abstract])) OR (PLWHIV[Title/Abstract])) OR (PLHIV[Title/Abstract])) OR (HIV-positive[Title/Abstract])) OR (HIV positive[Title/Abstract])) OR (HIV[MeSH Terms])) OR (AIDS[MeSH Terms])) AND (((((((((((((((((((Financial incentive[Title/Abstract]) OR (Cash incentive[Title/Abstract])) OR (Monetary[Title/Abstract])) OR (Non-cash incentive[Title/Abstract])) OR (None cash incentive[Title/Abstract])) OR (In-kind incentive[Title/Abstract])) OR (nonmonetary[Title/Abstract])) OR (non-monetary[Title/Abstract])) OR (Incentive[Title/Abstract])) OR (Motivation[Title/Abstract])) OR (Conditional cash transfer[Title/Abstract])) OR (CCT[Title/Abstract])) OR (Methods[Title/Abstract])) OR (Intervention[Title/Abstract])) OR (Financial Support[Title/Abstract])) OR (Motivation[MeSH Terms])) OR (monetary[MeSH Terms])) OR (Methods[MeSH Terms])) OR (Financial Support[MeSH Terms]))) AND ((((((((Retention[Title/Abstract]) OR (Retention in Care[Title/Abstract])) OR (Retention in Antiretroviral Therapy Programs[Title/Abstract])) OR (Retention in ART[Title/Abstract])) OR (Retained on treatment[Title/Abstract])) OR (Retained on HIV treatment[Title/Abstract])) OR (Retained on ART[Title/Abstract])) OR (Retention in Care[MeSH Terms]))) AND ((((((((((((((((((((((((((((((((((((((((((((((((((((((((((((((((((((((((((((((((((((((((((((((((((((((Angola[Title/Abstract]) OR (Benin[Title/Abstract])) OR (Botswana[Title/Abstract])) OR (Burkina Faso[Title/Abstract])) OR (Burundi[Title/Abstract])) OR (Cabo Verde[Title/Abstract])) OR (Cameroon[Title/Abstract])) OR (Central African Republic[Title/Abstract])) OR (Chad[Title/Abstract])) OR (Comoros[Title/Abstract])) OR (Democratic Republic of the Congo[Title/Abstract])) OR (Djibouti[Title/Abstract])) OR (Republic of the Congo[Title/Abstract])) OR (Congo[Title/Abstract])) OR (Cote d’Ivoire[Title/Abstract])) OR (Equatorial Guinea[Title/Abstract])) OR (Eritrea[Title/Abstract])) OR (Eswatini[Title/Abstract])) OR (Ethiopia[Title/Abstract])) OR (Gabon[Title/Abstract])) OR (Gambia[Title/Abstract])) OR (Ghana[Title/Abstract])) OR (Guinea[Title/Abstract])) OR (Guinea-Bissau[Title/Abstract])) OR (Kenya[Title/Abstract])) OR (Lesotho[Title/Abstract])) OR (Liberia[Title/Abstract])) OR (Madagascar[Title/Abstract])) OR (Malawi[Title/Abstract])) OR (Mali[Title/Abstract])) OR (Mauritania[Title/Abstract])) OR (Mauritius[Title/Abstract])) OR (Mozambique[Title/Abstract])) OR (Namibia[Title/Abstract])) OR (Niger[Title/Abstract])) OR (Nigeria[Title/Abstract])) OR (Rwanda[Title/Abstract])) OR (Sao Tome[Title/Abstract] AND Principe[Title/Abstract])) OR (Senegal[Title/Abstract])) OR (Seychelles[Title/Abstract])) OR (Sierra Leone[Title/Abstract])) OR (Somalia[Title/Abstract])) OR (South Africa[Title/Abstract])) OR (South Sudan[Title/Abstract])) OR (Sudan[Title/Abstract])) OR (Tanzania[Title/Abstract])) OR (Togo[Title/Abstract])) OR (Uganda[Title/Abstract])) OR (Zambia[Title/Abstract])) OR (Zimbabwe[Title/Abstract])) OR (sub-Saharan Africa[Title/Abstract])) OR (SSA[Title/Abstract])) OR (Africa South of the Sahara[Title/Abstract])) OR (Benin[MeSH Terms])) OR (botswana[MeSH Terms])) OR (burkina faso[MeSH Terms])) OR (burundi[MeSH Terms])) OR (Cabo Verde[MeSH Terms])) OR (cameroon[MeSH Terms])) OR (Central African Republic[MeSH Terms])) OR (chad[MeSH Terms])) OR (comoros[MeSH Terms])) OR (Democratic Republic of the Congo[MeSH Terms])) OR (djibouti[MeSH Terms])) OR (congo[MeSH Terms])) OR (cote d’ivoire[MeSH Terms])) OR (Equatorial Guinea[MeSH Terms])) OR (eritrea[MeSH Terms])) OR (Eswatini[MeSH Terms])) OR (ethiopia[MeSH Terms])) OR (gabon[MeSH Terms])) OR (gambia[MeSH Terms])) OR (ghana[MeSH Terms])) OR (Guinea[MeSH Terms])) OR (Guinea-Bissau[MeSH Terms])) OR (Kenya[MeSH Terms])) OR (lesotho[MeSH Terms])) OR (liberia[MeSH Terms])) OR (madagascar[MeSH Terms])) OR (malawi[MeSH Terms])) OR (Mali[MeSH Terms])) OR (mauritania[MeSH Terms])) OR (mauritius[MeSH Terms])) OR (mozambique[MeSH Terms])) OR (namibia[MeSH Terms])) OR (niger[MeSH Terms])) OR (nigeria[MeSH Terms])) OR (rwanda[MeSH Terms])) OR (Sao Tome and Principe[MeSH Terms])) OR (senegal[MeSH Terms])) OR (seychelles[MeSH Terms])) OR (sierra leone[MeSH Terms])) OR (somalia[MeSH Terms])) OR (south africa[MeSH Terms])) OR (South Sudan[MeSH Terms])) OR (sudan[MeSH Terms])) OR (tanzania[MeSH Terms])) OR (togo[MeSH Terms])) OR (uganda[MeSH Terms])) OR (zambia[MeSH Terms])) OR (zimbabwe[MeSH Terms])) OR (Africa South of the Sahara[MeSH Terms]))	1121	19-Apr-21
#4	(((((((((((((((((((((((((((((((((((((((((((((((((((((((((((((((((((((((((((((((((((((((((((((((((((((Angola[Title/Abstract]) OR (Benin[Title/Abstract])) OR (Botswana[Title/Abstract])) OR (Burkina Faso[Title/Abstract])) OR (Burundi[Title/Abstract])) OR (Cabo Verde[Title/Abstract])) OR (Cameroon[Title/Abstract])) OR (Central African Republic[Title/Abstract])) OR (Chad[Title/Abstract])) OR (Comoros[Title/Abstract])) OR (Democratic Republic of the Congo[Title/Abstract])) OR (Djibouti[Title/Abstract])) OR (Republic of the Congo[Title/Abstract])) OR (Congo[Title/Abstract])) OR (Cote d’Ivoire[Title/Abstract])) OR (Equatorial Guinea[Title/Abstract])) OR (Eritrea[Title/Abstract])) OR (Eswatini[Title/Abstract])) OR (Ethiopia[Title/Abstract])) OR (Gabon[Title/Abstract])) OR (Gambia[Title/Abstract])) OR (Ghana[Title/Abstract])) OR (Guinea[Title/Abstract])) OR (Guinea-Bissau[Title/Abstract])) OR (Kenya[Title/Abstract])) OR (Lesotho[Title/Abstract])) OR (Liberia[Title/Abstract])) OR (Madagascar[Title/Abstract])) OR (Malawi[Title/Abstract])) OR (Mali[Title/Abstract])) OR (Mauritania[Title/Abstract])) OR (Mauritius[Title/Abstract])) OR (Mozambique[Title/Abstract])) OR (Namibia[Title/Abstract])) OR (Niger[Title/Abstract])) OR (Nigeria[Title/Abstract])) OR (Rwanda[Title/Abstract])) OR (Sao Tome[Title/Abstract] AND Principe[Title/Abstract])) OR (Senegal[Title/Abstract])) OR (Seychelles[Title/Abstract])) OR (Sierra Leone[Title/Abstract])) OR (Somalia[Title/Abstract])) OR (South Africa[Title/Abstract])) OR (South Sudan[Title/Abstract])) OR (Sudan[Title/Abstract])) OR (Tanzania[Title/Abstract])) OR (Togo[Title/Abstract])) OR (Uganda[Title/Abstract])) OR (Zambia[Title/Abstract])) OR (Zimbabwe[Title/Abstract])) OR (sub-Saharan Africa[Title/Abstract])) OR (SSA[Title/Abstract])) OR (Africa South of the Sahara[Title/Abstract])) OR (Benin[MeSH Terms])) OR (botswana[MeSH Terms])) OR (burkina faso[MeSH Terms])) OR (burundi[MeSH Terms])) OR (Cabo Verde[MeSH Terms])) OR (cameroon[MeSH Terms])) OR (Central African Republic[MeSH Terms])) OR (chad[MeSH Terms])) OR (comoros[MeSH Terms])) OR (Democratic Republic of the Congo[MeSH Terms])) OR (djibouti[MeSH Terms])) OR (congo[MeSH Terms])) OR (cote d’ivoire[MeSH Terms])) OR (Equatorial Guinea[MeSH Terms])) OR (eritrea[MeSH Terms])) OR (Eswatini[MeSH Terms])) OR (ethiopia[MeSH Terms])) OR (gabon[MeSH Terms])) OR (gambia[MeSH Terms])) OR (ghana[MeSH Terms])) OR (Guinea[MeSH Terms])) OR (Guinea-Bissau[MeSH Terms])) OR (Kenya[MeSH Terms])) OR (lesotho[MeSH Terms])) OR (liberia[MeSH Terms])) OR (madagascar[MeSH Terms])) OR (malawi[MeSH Terms])) OR (Mali[MeSH Terms])) OR (mauritania[MeSH Terms])) OR (mauritius[MeSH Terms])) OR (mozambique[MeSH Terms])) OR (namibia[MeSH Terms])) OR (niger[MeSH Terms])) OR (nigeria[MeSH Terms])) OR (rwanda[MeSH Terms])) OR (Sao Tome and Principe[MeSH Terms])) OR (senegal[MeSH Terms])) OR (seychelles[MeSH Terms])) OR (sierra leone[MeSH Terms])) OR (somalia[MeSH Terms])) OR (south africa[MeSH Terms])) OR (South Sudan[MeSH Terms])) OR (sudan[MeSH Terms])) OR (tanzania[MeSH Terms])) OR (togo[MeSH Terms])) OR (uganda[MeSH Terms])) OR (zambia[MeSH Terms])) OR (zimbabwe[MeSH Terms])) OR (Africa South of the Sahara[MeSH Terms])	424491	19-Apr-21
#3	(((((((Retention[Title/Abstract]) OR (Retention in Care[Title/Abstract])) OR (Retention in Antiretroviral Therapy Programs[Title/Abstract])) OR (Retention in ART[Title/Abstract])) OR (Retained on treatment[Title/Abstract])) OR (Retained on HIV treatment[Title/Abstract])) OR (Retained on ART[Title/Abstract])) OR (Retention in Care[MeSH Terms])	186421	19-Apr-21
#2	((((((((((((((((((Financial incentive[Title/Abstract]) OR (Cash incentive[Title/Abstract])) OR (Monetary[Title/Abstract])) OR (Non-cash incentive[Title/Abstract])) OR (None cash incentive[Title/Abstract])) OR (In-kind incentive[Title/Abstract])) OR (nonmonetary[Title/Abstract])) OR (non-monetary[Title/Abstract])) OR (Incentive[Title/Abstract])) OR (Motivation[Title/Abstract])) OR (Conditional cash transfer[Title/Abstract])) OR (CCT[Title/Abstract])) OR (Methods[Title/Abstract])) OR (Intervention[Title/Abstract])) OR (Financial Support[Title/Abstract])) OR (Motivation[MeSH Terms])) OR (monetary[MeSH Terms])) OR (Methods[MeSH Terms])) OR (Financial Support[MeSH Terms])	6161803	19-Apr-21
#1	(((((((((((HIV[Title/Abstract]) OR (Human immunodeficiency virus[Title/Abstract])) OR (AIDS[Title/Abstract])) OR (Acquired Immunodeficiency Syndrome[Title/Abstract])) OR (HIV&AIDS[Title/Abstract])) OR (People Living with HIV[Title/Abstract])) OR (PLWHIV[Title/Abstract])) OR (PLHIV[Title/Abstract])) OR (HIV-positive[Title/Abstract])) OR (HIV positive[Title/Abstract])) OR (HIV[MeSH Terms])) OR (AIDS[MeSH Terms])	438027	19-Apr-21

### 2.5. Selection process

SM and JG independently applied the adapted eligibility assessment form to retrieve the studies that responded to the research question**: What interventions are used to improve early retention of patients in ART programmes in SSA?.** The population included all gender, ages and ethnicity of HIV positive patients in SSA. The exposure/intervention included all interventions targeted at improving early retention of patients on ART programmes. This being a qualitative systematic review, all study designs were included in the review. The study outcome was retention on ART programmes at 6 months. We followed the PRISMA guidelines to report study selection in the PRISMA flow chart. The authors SM and JG independently screened the studies using the adapted eligibility assessment form to decide on the studies to include in the review and those to exclude and captured the reasons for exclusion. The authors YK and SK reviewed and approved the screening processes.

### 2.6. Data collection process

The lead author (SM) developed the data extraction tool in excel based on the Cochrane Data Collection form for intervention reviews for RCT and non-RCTs template [18]. The adapted tool enable compliance to Methodological Expectations of Cochrane Intervention Reviews (MECIR) standards for collecting and reporting information about studies for review, and analysing their results [20]. Both SM and JG independently reviewed the full articles and summarised the data as per the data extraction tool. The report was then reviewed by the co-investigators YK and SK.

### 2.7. Data items

The adapted tool included the following elements: 1) Author(s) and date of publication, 2) Study title, 3) The intervention and control, 4) Country of implementation, 5) Target population, 6) Study design, 7) Outcome of measure, 8) comments. See Table 3 for the structure of the adapted tool.

**Table 3:** Description of full text studies included in the review.

#	Author & Year	Title	Intervention and Control	Country of implementation	Target population	Study design	Outcome measure	Comments
**1**	**Asieba, I. O., et al. (2021)**	**Antiretroviral therapy in community pharmacies—Implementation and outcomes of a differentiated drug delivery model in Nigeria.**	**Community pharmacy-based ART refill model for stable clients. Stable patients devolved to community pharmacies for routine refills at a service fee of $3 per refill visit**	**Nigeria**	**HIV positive adults**	**Retrospective cohort study**	**Proportion of patients retained on treatment at 6 months**	**Retention rate was 98%. This community pharmacy ART refill model of differentiated care demonstrated excellent clinical outcomes of retention and viral suppression**
**2**	**Khumalo, P. N., et al. (2020)**	**The Cascade of Care from Routine Point-of-Care HIV Testing at Birth: Results From an 18-Months Pilot Program in Eswatini**	**Point-of-care HIV birth testing**	**Eswatini/Swaziland**	**Infants testing HIV-positive at birth**	**Cohort study**	**Proportion of ART-initiated infants on ART at 6 months after initiation**	**POC birth testing demonstrated high retention rate of 84%**
**3**	**Herce, M. E., et al. (2020)**	**Universal test-and-treat in Zambian and South African correctional facilities: a multisite prospective cohort study**	**Universal test-and-treat (UTT) intervention in correctional facilities. Treatment as Prevention (TasP) involving same-day ART initiation**	**South Africa and Zambia**	**Incarcerated individuals aged 18 years or older, with new or previously diagnosed HIV**	**Prospective Cohort study**	**Retained in care at 6 months defined as any documented clinical, pharmacy, or laboratory visit occurring 84 days or more after ART initiation among all participants who remained incarcerated at the facility at 6 months**	**95% retained in care. UTT implementation can achieve high levels of retention in care among incarcerated people with HIV that are comparable to those observed in community settings**
**4**	**Fahey, C. A., et al. (2020)**	**Financial incentives to promote retention in care and viral suppression in adults with HIV initiating antiretroviral therapy in Tanzania: a three-arm randomised controlled trial**	**Arm 1: Cash incentive of US$4·50. Arm 2: Cash incentive of US$10·00. *Control*: Usual care**	**Tanzania**	**Adults aged 18 years or older with HIV who had started ART within the past 30 days**	**RCT**	**Proportion retained in care at 6 months**	**83% and 86% in the smaller and larger incentive groups respectively and 73% in control group were retained in care. Small financial incentives delivered using mHealth can improve retention in care in adults starting HIV treatment**
**5**	**Ahmed, I., et al. (2020)**	**Effectiveness of same-day antiretroviral therapy initiation in retention outcomes among people living with human immunodeficiency virus in Ethiopia: empirical evidence**	***Cohort 1*: PLHIV who started ART on same-day following diagnosis verses *Cohort 2*: PLHIV who started ART >7 days after HIV diagnosis.**	**Ethiopia**	**Newly initiated on ART aged 15 years and above**	**Multicentre retrospective cohort study**	**Proportion of PLHIV known to be alive and receiving ART at 6 months**	**82% and 89.4% retained in the same-day and > 7 days ART initiation groups respectively. Compared to the > 7 days group, the unadjusted and ad- justed RR of 6-months retention for same-day group were 0.92 (95% CI: 0.87, 0.97; p < 0.001) and 0.89 (95% CI: 0.87, 0.90; p < .001), respectively**
**6**	**Fayorsey, R. N., et al. (2019)**	**Effectiveness of a Lay Counsellor-Led Combination Intervention for Retention of Mothers and Infants in HIV Care: A Randomized Trial in Kenya**	**Combination intervention of Lay counsellor called *Mama Mshauri* who provided 1) individualized PMTCT health education using a standardized flip chart during home and clinic visits; 2) retention and adherence support; 3) phone and SMS appointment reminders; and 4) follow-up and tracking for missed clinic visits. *Control*: Routine ANC, delivery, postpartum, and PMTCT care as per Kenyan national guidelines by health facility MCH staff.**	**Kenya**	**Pregnant HIV-positive women and their infants**	**Individualized RCT**	**Mother–infant attrition defined as proportion of mother–infant pairs not retained in the clinic at 6 months postpartum because of mother or infant death, or lost to follow-up**	**LTFU was defined as no documented clinic attendance at 6 months postpartum in the 3 months prior or after the 6-month scheduled visit. 81.2% and 71.8% retention rate in the intervention and SOC arms respectively. Provision of a combination intervention by lay counsellors can decrease attrition along the PMTCT cascade in low-resource settings**
**7**	**Abrams, E. J., et al. (2019)**	**Impact of universal antiretroviral therapy for pregnant and postpartum women on antiretroviral therapy uptake and retention.**	**Change from CD4+-guided ART eligibility (’Option A’), to Option B+ (universal antiretroviral therapy)**	**Eswatini/Swaziland**	**HIV positive Pregnant women**	**A stepped-wedge evaluation**	**Postnatal retention defined as proportion of women attending clinic within 84 days of 6-months postpartum**	**Retention was higher under Option B+ (53%) vs. Option A (24%). Adjusting for age, gestational age, previous HIV diagnosis, and CD4+, Option B+ women were significantly more likely to be retained postnatally (aRR 2.11; 95% CI 1.79–2.49) compared with Option A. Universal ART resulted in substantial increases in pregnant women retained in care through 6 months postpartum**
**8**	**Mody, A., et al. (2018)**	**Estimating the real-world effects of expanding antiretroviral treatment eligibility: Evidence from a regression discontinuity analysis in Zambia.**	**Expanding treatment eligibility from 350 to 500 cells/μL and instituting treatment for all pregnant and breastfeeding women under Option B+**	**Zambia**	**HIV positive adults >15 years old newly enrolled in HIV**	**Regression Discontinuity Design**	**Retention in care at 6 months defined as having made at least one visit between 3- and 9-months post-enrolment**	**4.1% absolute increase in retention in care at 6 months (95% CI, 1.6%–6.7%, p = 0.0014). Guidelines raising the CD4 threshold for treatment from 350 to 500 cells/μL were associated with a rapid rise in retention among newly treatment-eligible patients, without negatively impacting patients with lower CD4 levels**
**9**	**Izudi, J., et al. (2018)**	**Retention of HIV-Positive adolescents in care: A quality improvement intervention in mid-western Uganda**	**A quality improvement (QI) project implementing improvement changes with Plan-Do-Study-Act (PDSA). It involved the following: 1) Retrospective review of HIV data for retention levels; 2) Conducted 1 hour CME session on basic concepts of QI; 3) Reconstituted the health facility QI Team with a member from each department and formed departmental QI teams to address performance gaps at departmental level; 4) Supported the HIV QI Team to start a QI project to address the low retention of HIV-positive adolescents in care through Problem Identification, Root Cause Analysis, Developing and Prioritizing Solutions and Implementation of Improvement Changes.**	**Uganda**	**HIV positive adolescents**	**Quality Improvement design**	**Retention at 6 months**	**Retention rate of 96.8%. Context specific, integrated, adolescent-centred interventions implemented using QI significantly improved retention in Mid-Western Uganda**
**10**	**Graves, J. C., et al. (2018)**	**Impact of a Family Clinic Day intervention on paediatric and adolescent appointment adherence and retention in antiretroviral therapy: a cluster randomized controlled trial in Uganda**	**Differentiated care model called Family Clinic Day (FCD) involving the following components: 1) A family‐centred appointment scheduling; 2) Health education led by an expert client and 3) Patient flow where children, adolescents and their families were prioritized for care over other patients. *Control*: Standard of care**	**Uganda**	**Paediatric and adolescent patients aged between 19 months and 19 years, and active in care during the first three months of the study**	**RCT**	**Proportion retained in care at 6 months**	**aOR 1.11; 90% CI 0.63‐1.97, p = 0.75. The FCD did not improve retention**
**11**	**Stevens, W. S., et al. (2017)**	**Multidisciplinary Point-of-Care Testing in South African Primary Health Care Clinics Accelerates HIV ART Initiation but Does Not Alter Retention in Care**	**Point-of-care testing where participants had phlebotomy and POCT immediately on-site using Pima CD4 to assess ART eligibility followed by haematology, chemistry, and tuberculosis screening with the goal of receiving same-day adherence counselling and treatment initiation. *Control*: SOC participants were venesected and specimens referred to the laboratory with patient follow-up as per algorithm**	**South Africa**	**HIV positive adults**	**RCT**	**Proportion retained in care at 6 months**	**The proportion of patients in care and on ART was similar for both arms at 6 months (47 vs. 50%) (aPR 0.96; 95% CI: 0.79 to 1.16)**
**12**	**Sam-Agudu, N. A., et al. (2017)**	**The impact of structured mentor mother programs on 6-month postpartum retention and viral suppression among HIV-positive women in rural Nigeria: A prospective paired cohort study**	**Structured Peer support through mentor mothers (MMs). MMs are HIV-positive, PMTCT-experienced women who counsel less-experienced peers for optimal PMTCT outcomes. They offered structured peer support consisting of intensive baseline training, a detailed scope of work, close mentoring and supervision by a designated Mentor**	**Nigeria**	**HIV positive pregnant women**	**Cohort study**	**Postpartum retention at 6 months defined as participants attending 3 of 6 expected monthly visits were considered retained**	**MM support was associated with higher odds of retention than routine PS (adjusted odds ratio = 5.9, 95% confidence interval: 3.0 to 11.6). Structured PS significantly improved postpartum PMTCT retention**
			**Mother supervisor, a standardized logbook for documentation of field activities, and periodic performance evaluations. This was compared to loosely-organized, less-supervised routine peer support**					
**13**	**Oyeledun, B., et al. (2017)**	**The Effect of a Continuous Quality Improvement Intervention on Retention-In-Care at 6 Months Postpartum in a PMTCT Program in Northern Nigeria: results of a Cluster Randomized Controlled Study**	**CQI Teams composed and agreed on priority areas for improvement and then used the Plan-Do-Study-Act model to formulate action plans, agree on indicators, collate and analyse facility data, and thereby test ideas for improving these specific areas. CQI coaches visited every 2 weeks to guide implementation of change ideas, including how to measure the process and indicators of change ideas being tested, plot run-charts and provide structured assessment and response tools; technical assistance was also provided by phone. *Control arm*: Received routine support as per the Nigeria MOH guidelines**	**Nigeria**	**HIV positive pregnant women with gestational age of 34 weeks or less**	**RCT**	**Postpartum retention at 6 months defined as women who visit (+ or -30 days) and did not miss any previous scheduled visit by more than 30 days starting from ANC booking**	**44% vs. 41% [ARR = 1.08; 95% CI:0.78 to 1.49] retention rate among intervention and control groups. CQI as implemented in this study did not differ across study arms in the rates of retention**
**14**	**Kiragga, A. N., et al. (2016)**	**Implementation and Operational Research: Impact of Nurse-Targeted Care on HIV Outcomes Among Immunocompromised Persons: A Before-After Study in Uganda**	***Nurse counsellor cohort*: 1 additional nurse per clinic was hired (nurse counsellor cohort) to identify new patients, expedite ART initiation, and trace those who were lost to follow-up for 6 months. Routine care cohort: Received Standard of care for all HIV positive persons as per Ugandan HIV guidelines**	**Uganda**	**HIV positive adults**	**Pre and post**	**Proportion retained in care at 6 months**	**62% vs 76% retention rates among those in routine care and in the nurse counsellor cohort respectively. Implementation of targeted nurse-led care of severely immunocompromised HIV-infected patients in public outpatient health care facilities resulted in increased retention.**
**15**	**Chan, A. K., et al. (2016)**	**Same day HIV diagnosis and antiretroviral therapy initiation affects retention in Option B+ prevention of mother-to-child transmission services at antenatal care in Zomba District, Malawi**	**Option B+ service delivery ‘‘model of care” described in terms of the degree of integration of ANC, HTC and ART. *Model 1*: Full integration of HTC and ART initiation at ANC. *Model 2*: Integration of HTC only into ANC services with subsequent referral to an existing ART clinic for treatment initiation**	**Malawi**	**HIV positive pregnant women**	**Cohort study**	**Postpartum retention at 6 months**	**78% vs. 92% p = 0.001 retention in Model 1 compared to Model 2 sites. ANC clinics provide integrated HTC and ART initiation services (Model 1) had lower retention on ART compared to model 2 facilities**
**16**	**Kim, M. H., et al. (2015)**	**Implementation and operational research: the impact of option B+ on the antenatal PMTCT cascade in Lilongwe, Malawi**	**Pre Option B+ (October 2009 -March 2011): Opt-out HIV antibody testing at ANC; CD4+ cell count testing; If WHO stage 3/4 or CD4+ cell count, 350 cell/mm3,FDC of d4T-3TC-NVP twice daily for life; If CD4+ cell count $350 cell/mm3, AZT from 28-wk gestation to delivery + sdNVP at delivery + FDC of AZT + 3TC twice daily starting at delivery for 7 days; sdNVP + AZT for 1 week; DNA-PCR of infant dry blood spots recommended at 6 weeks of age; Case management by CHW including facility- and community-based ARV adherence supervision, counselling, and follow-up visits. Other CHW responsibilities include facility-based health talks, nutritional assessments at clinic visits, and HIV testing and counselling. Each CHW follows an average of 35–50 patients. *Post Option B+*: No CD4+ cell count testing; FDC of TDF-3TC-EFV once daily for life for all pregnant women found to be HIV+; Pregnant women offered ART on the same day HIV status is ascertained; Daily nevirapine for 6 weeks. **The rest of the interventions are similar to pre-option B+****	**Malawi**	**HIV positive pregnant women**	**Pre and post**	**Postpartum retention at 6 months**	**Retention was higher among the post option B+ group**
**17**	**Mosoko, J. J., et al. (2011)**	**Retention in an antiretroviral therapy programme during an era of decreasing drug cost in Limbe, Cameroon**	***Cohort 1*: patients who initiated ART before 1 October 2004; *Cohort 2*: patients who initiated ART on or after 1 October 2004 when the prices of ARV drugs declined substantially to US $5.50 and US$12.80 per month for first-and second-line treatment regimens, respectively.**	**Cameroon**	**HIV positive adults**	**Pre and post**	**Retention at 6 months**	**The probabilities of remaining alive and in care were 0.66 (95% CI 0.64–0.68) at six months. Not significantly different before and after price reduction. Reducing the cost of ART did not change retention in care (HR 1.1; 95% CI 0.9–1.2)**

### 2.8. Study risk of bias assessment

The risk of bias was assessed using RoB2: A revised Cochrane risk of bias tool for randomized trials [21] and the Newcastle-Ottawa Scale (NOS) for observational studies [22]. Depending on whether the randomized trials were either parallel group or cluster, we used the approapriate RoB2 assessment form to determine the risk of bias. The parallel group randomized trials were assessed based on five domains of 1) Randomisation process, 2) Deviations from intended interventions, 3) Missing outcome data, 4) Measurement of the outcome and 5) Selection of the reported result. The cluster group randomized trials were assessed on the same five domains in addition to the timing of identification or recruitment of participants. An observational study could be awarded a maximum of one star for each numbered item within the Selection and Outcome/Exposure categories and a maximum of two stars could be awarded for Comparability. Each star was scored as one (1) while no star was scored as zero (0). The risk of bias assessment for the observational studies was based on the total quality scores from the three categories, with the total maximum score being 9. A study with score from 7–9 was considered high quality (low risk of bias), those with score from 4–6 were considered as having some concerns and a score from 0–3 were considered high risk of bias. Depending on the study design, SM and JG independently scored the peer reviewed studies included in the final review using the RoB2 and the NOS tools and resolved any discrepancies (differences in the scores) through discussion and concensus.

### 2.9. Certainity assessment

Retrieved published and unpublished studies were assessed for inclusion using their title and abstract. Then a full review of articles for quality assessment was done before selection for final review. SM and JG independently assessed the articles for inclusion in the review. Any discrepancy which arose between the authors on the review process was solved through discussion and concensus.

## 3. Results

### 3.1. Study selection

**Fig 1:**
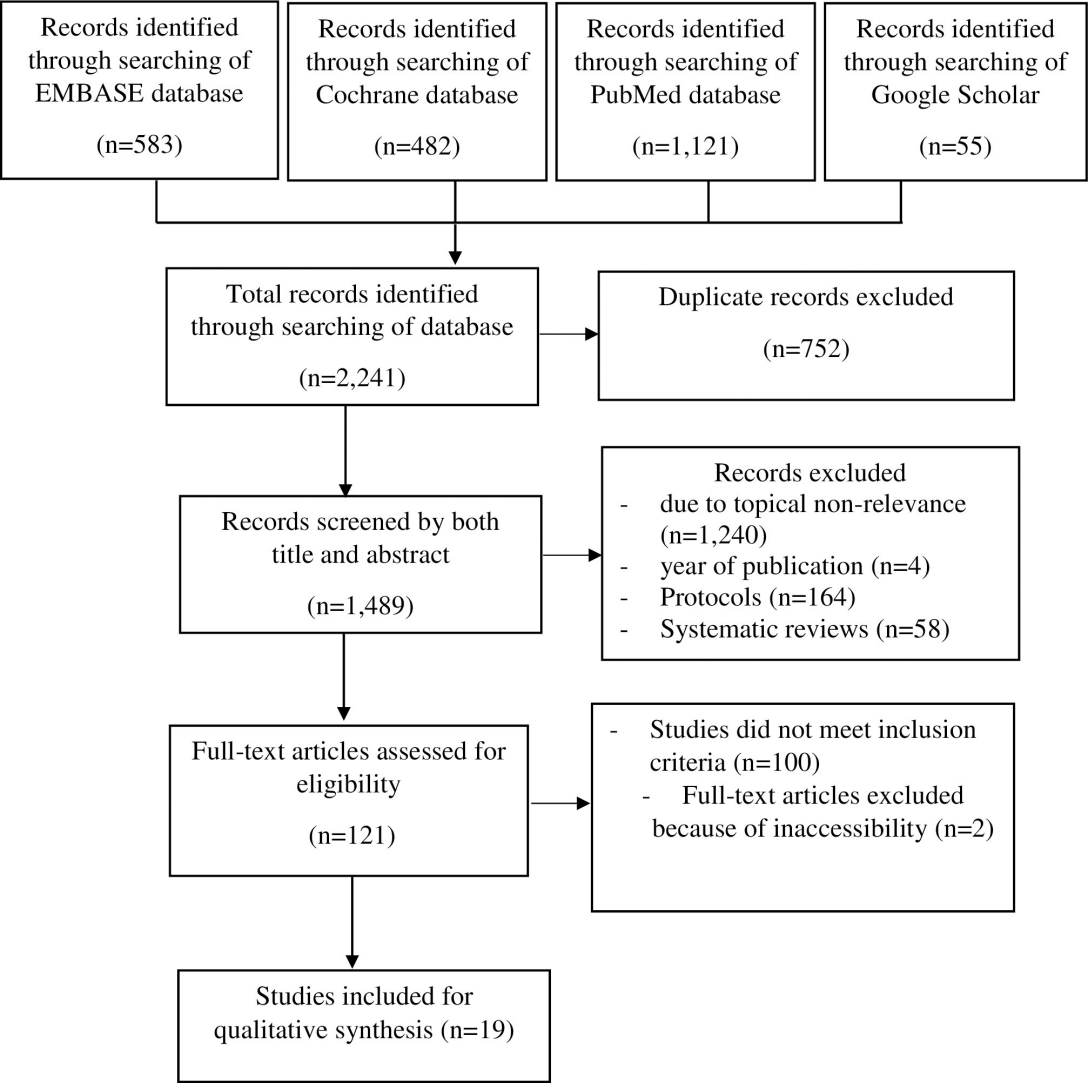
PRISMA flow chart. Shows the literature search and selection process used in this study.

A total of 2,241 articles were identified from searches of electronic databases and review of article references, of which 752 duplicates were dropped, 1,142 were excluded due to topical non-relevance, four (4) excluded as they were published before the year 2000 which was the cut-off year, 164 and 58 study protocols and systematic reviews were excluded respectively. We further screened the remaining 121 articles for relevance in both the title and abstract, dropping 100 articles which were documenting interventions targeted at improving retention earlier or later than six (6) months from first ART initiation. We further dropped two (2) articles that were not fully accessible and proceeded with 19 articles for full screening as they met the inclusion criteria and were considered eligible for this review (Fig 1).

### 3.2. Study characteristics

**Fig 2:**
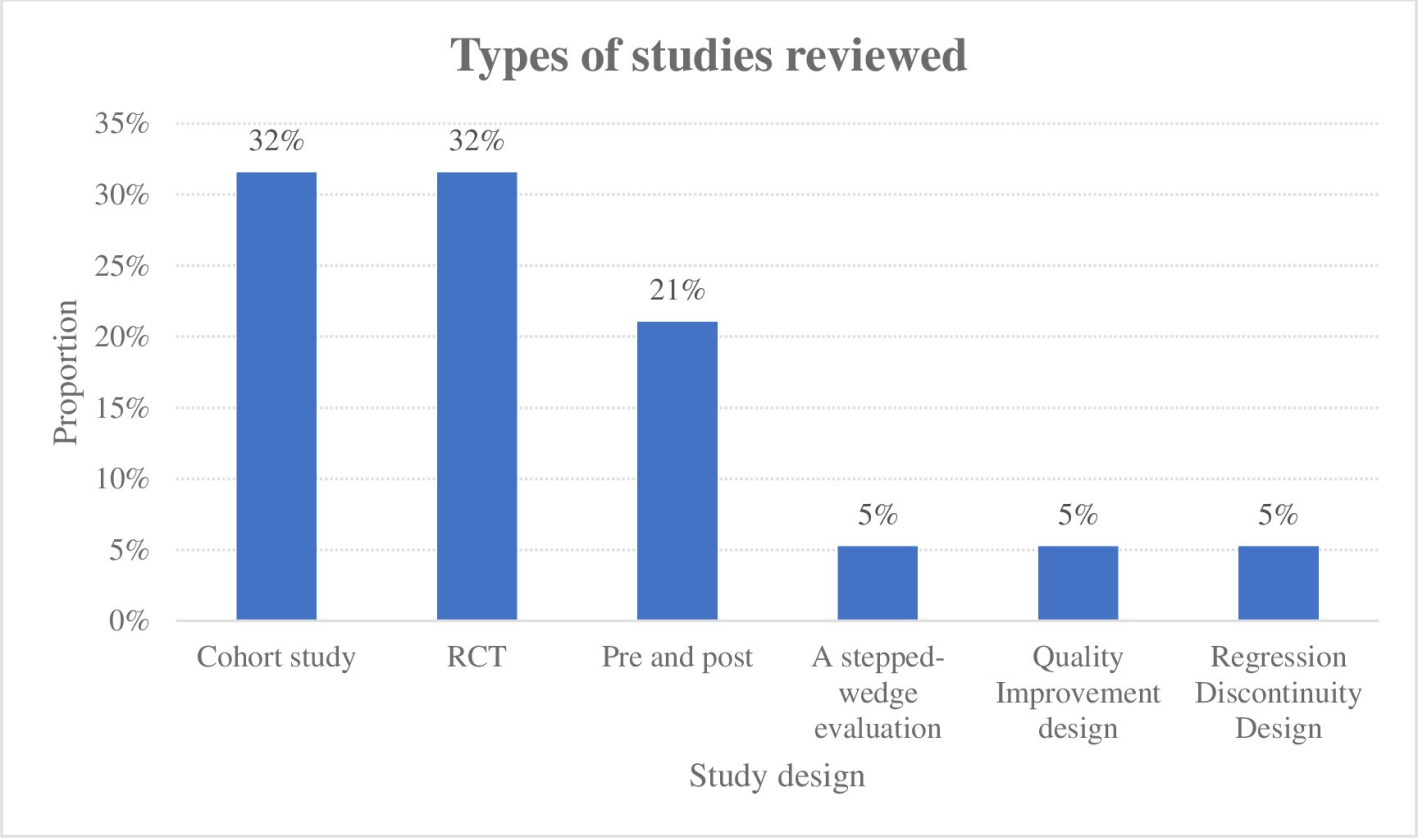
Distribution of study designs used in the included studies. Graph summarizing the designs of studies reviewed.

Of the studies included for systematic review, 32% (n = 6) were randomized control trials [23–28], 32% (n = 6) were either prospective or retrospective cohort studies [29–34], 21% (n = 4) were pre and post/before and after studies [35–38] and the rest were quality improvement design [39], regression discontinuity design [40] and a stepped-wedge evaluation [41] (Fig 2).

The studies reviewed were conducted in 11 SSA countries: Uganda (n = 3) [28, 36, 39], Nigeria (n = 3) [24, 29, 33], South Africa (n = 2) [23, 25], Malawi (n = 2) [34, 37], Swaziland (n = 2) [30, 41], Kenya (n = 1) [27], Zambia (n = 1) [40], Tanzania (n = 1) [26], Ethiopia (n = 1) [32], Cameroon (n = 1) [38], Botswana (n = 1) [35] and South Africa and Zambia (n = 1) [31].

**Fig 3:**
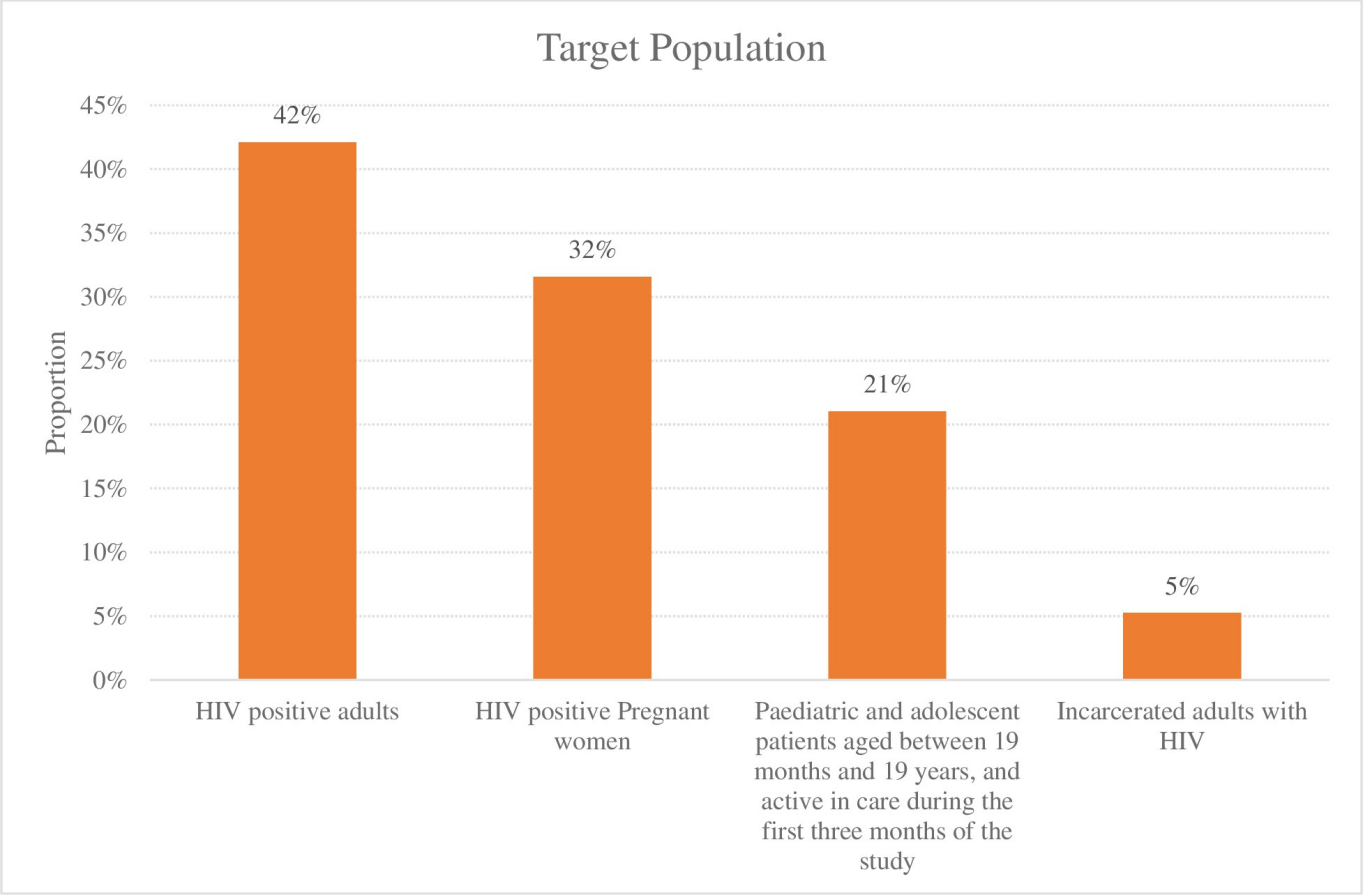
Target population in the reviewed studies. Summarizes the target population in the studies reviewed.

The documented interventions targeted HIV positive adults (42%, n = 8) [23, 25, 26, 29, 35, 36, 38, 40], HIV positive pregnant women (32%, n = 6) [24, 27, 33, 34, 37, 41], paediatrics and adolescents (21%, n = 4) [28, 30, 32, 39] and incarcerated adults with HIV [31] (Fig 3).

Characteristics of the included studies are summarised in Tables 3 and 4. Seventeen studies were published in peer reviewed journals and two were conference abstracts.

**Table 4:** Description of conference abstracts included in the review.

#	Author & Year	Title	Intervention and Control	Country of implementation	Target population	Study design	Outcome measure	Comments
1	Violette, L., et al. (2020)	Community-based antiretroviral therapy delivery associated with viral suppression and retention in care in South Africa.	Community based ART delivery programme (CCMDD). Included point‐of‐care VL testing and task‐shifting to an enrolled nurse. *Control*: standard laboratory VL testing	South Africa	HIV positive adults	RCT	Proportion of patients retained on treatment at 6 months	Retention in care (RR = 1.08 p = 0.017). Among clinically stable participants, those enrolled in the CCMDD programme had higher rates of retention in care, indicating that the community‐based ART delivery model did not negatively impact HIV care outcomes.
2	Lebelonyane, R., et al. (2018)	Fast-track ART initiation in Botswana is associated with high rates of ART initiation, retention in care, and virological suppression	“fast-track” ART initiation at the first clinic visit versus pre- fast track ART initiation	Botswana	HIV-infected individuals not on ART	Pre and post	Proportion retained in care after six months on ART	93% retention rates among the pre and post groups. Retention in care after 6 months on ART was same in both groups

### 3.3. Risk of bias in studies

We conducted a risk of bias assessment for the seventeen peer reviewed studies in this review. Majority of the studies were observational (n = 12) while three were individual randomized control trial [23, 26, 27] and two cluster randomized trials [24, 28]. Of the seventeen peer reviewed studies assessed for quality of the evidence, the majority (n = 11) were rated as having low risk of bias, while six (6) studies were classified as having some concerns. All the five (5) randomized control trials were overally categorised as having low risk of bias though with some concerns in two areas, namely: Deviations from intended interventions and Measurement of the outcome in two of the studies. Of the twelve observational studies, six (6) were rated as having low risk of bias and the other six (6) noted with some concerns with the main quality issues related to non description of the derivation of the non exposed cohorts, non demonstration that the outcome of interest was not present at start of the study, lack of clarity on the variables controlled for during data analysis and no statements on adequacy in follow-up of cohorts. Table 5 shows the quality assessment report of the published peer reviewed studies.

**Table 5:** Quality assessment of published peer reviewed studies.

Author	Study design	Selection				Comparability		Outcome/Exposure			Total Quality score*	Overall Bias
Asieba/2021	Retrospective cohort	1	0	1	1	0	0	0	1	1	5	Some concerns
Khumalo/2020	Prospective cohort	1	0	1	1	0	0	1	1	1	6	Some concerns
Herce/2020	Prospective cohort	1	1	1	1	1	1	1	1	1	9	Low risk of bias
Ahmed/2020	Retrospective cohort	1	1	1	1	1	1	1	1	1	9	Low risk of bias
Abrams/2019	Stepped-wedge evaluation	1	1	1	1	1	1	1	1	1	9	Low risk of bias
Mody/2018	Regression Discontinuity	1	1	1	1	1	1	1	1	1	9	Low risk of bias
Izudi/2018	Observational study	1	0	1	1	0	0	1	1	1	6	Some concerns
Sam-Agudu/2017	Prospective cohort	1	1	1	1	1	1	1	1	1	9	Low risk of bias
Kiragga/2016	Before-After	1	0	1	0	1	0	1	1	0	5	Some concerns
Chan/2016	Retrospective cohort	1	1	1	1	1	1	1	1	1	9	Low risk of bias
Kim/2015	Before-After	1	0	1	0	1	0	1	1	0	5	Some concerns
Mosoko/2011	Before-After	1	0	1	1	1	0	1	1	0	6	Some concerns
Fahey/2020	Individual RCT	Randomization process- Low risk of bias										Low risk of bias
		Deviations from intended interventions- Low risk of bias										
		Missing outcome data- Low risk of bias										
		Measurement of the outcome- Low risk of bias										
		Selection of the reported result- Low risk of bias										
Fayorsey/2019	Individual RCT	Randomization process- Low risk of bias										Low risk of bias
		Deviations from intended interventions- Some concerns										
		Missing outcome data- Low risk of bias										
		Measurement of the outcome- Low risk of bias										
		Selection of the reported result- Low risk of bias										
Stevens/2017	Individual RCT	Randomization process- Low risk of bias										Low risk of bias
		Deviations from intended interventions- Low risk of bias										
		Missing outcome data- Low risk of bias										
		Measurement of the outcome- Low risk of bias										
		Selection of the reported result- Low risk of bias										
Graves/2018	Cluster RCT	Randomization process- Low risk of bias										Low risk of bias
		Timing of identification or recruitment of participants- Low risk of bias										
		Deviations from intended interventions- Low risk of bias										
		Missing outcome data- Low risk of bias										
		Measurement of the outcome- Low risk of bias										
		Selection of the reported result- Low risk of bias										
Oyeledun/2017	Cluster RCT	Randomization process- Low risk of bias										Low risk of bias
		Timing of identification or recruitment of participants- Low risk of bias										
		Deviations from intended interventions- Low risk of bias										
		Missing outcome data- Low risk of bias										
		Measurement of the outcome- Some concerns										
		Selection of the reported result- Low risk of bias										

*Total Quality Score categories are: Score of 7–9 (Low risk of bias); Score of 4–6 (Some concerns) and Score of 0–3 (high risk of bias).

### 3.4. Interventions to improve early retention in ART

Interventions identified as a tempting to improve early retention of patients on ART were categorised into the following broad areas: 1) Health system interventions, 2) Patient centered approaches, 3) Behaviour interventions and support and 4) Incentives.

#### 3.4.1. Health system interventions

*3*.*4*.*1*.*1*. *Universal test-and-treat*. Adoption of universal test-and-treat policy in Zambian and South African correctional facilities showed improved retention of incarcerated HIV positive individuals in care at six (6) months [31]. The multisite, mixed methods, implementation research study with a prospective cohort component showed that 95% of participants who remained incarcerated were retained in the on-site treatment programme at 6 months. Two of the studies, one in Swaziland [41] and the other in Malawi [37] assessed the impact of implementing option B+ on the antenatal Prevention of Mother to Child Transmission (PMTCT) cascade. The stepped-wedge evaluation in Swaziland showed that postnatal retention among HIV positive pregnant women was higher under option B+ (53%) compared to before implementation of option B+ policy (option A) (24%). Adjusting for age, gestational age, previous HIV diagnosis, and CD4+, Option B+ women were significantly more likely to be retained postnatally (adjusted Risk Ratio (aRR) 2.11; 95% Confidence Interval (CI) 1.79–2.49) compared with Option A. Equally in Malawi, a significantly greater proportion of HIV positive pregnant women were retained through delivery pre-option B+ (51.1%) vs post option B+ (65%) p< 0.0001 [37]. A regression discontinuity design study in Zambia estimated the the real- world effects of expanding antiretroviral treatment eligibility from 350 to 500 cells/ul among HIV positive adults found a 4.1% absolute increase in retention in care at 6 months (95% CI, 1.6%–6.7%, p = 0.0014). Without adversely affecting patients with lower CD4 levels, guidelines raising the CD4 cut-off for treatment from 350 to 500 cells/μL were associated with a rapid rise in retention among newly treatment-eligible patients [40].

*3*.*4*.*1*.*2*. *Integration*. Full integration of HIV Testing and Counseling (HTC) and ART initiation at antenatal care (ANC) services yielded lower postpartum retention at six (6) months compared with integration of HTC only into ANC services with subsequent referral to an existing ART clinic for treatment initiation (78% vs. 92% p = 0.001) [34].

*3*.*4*.*1*.*3*. *Reduced drug cost*. Cameroon initiated a substantial reduction in ART cost on 1st October 2004. Mosoko, J. J., et al, through a pre and post study assessed the impact of this event and other factors on enrolment and retention in care among HIV-infected patients initiating ART from February 2002 to December 2005 at the single ART clinic serving the Southwest Region in Limbe, Cameroon [38]. The study found that the probabilities of remaining alive and in care were 0.66 (95% CI 0.64–0.68) at six (6) months and not significantly different before and after price reduction.

#### 3.4.2. Patient centered approaches

*3*.*4*.*2*.*1*. *Fast track ART initiation*. In Ethiopia, a multicenter retrospective cohort study assessed the effectiveness of same-day antiretroviral therapy initiation in retention outcomes among PLHIV. Cohort 1 were PLHIV who started ART on same-day following diagnosis verses Cohort 2 who were PLHIV who started ART more than 7 days after HIV diagnosis. The study found that 82% and 89.4% of PLHIVs were retained in the same-day and more than 7 days ART initiation groups respectively. In comparison to the more than 7 days group, the unadjusted and adjusted Risk Ratio (aRR) of 6-months retention for same-day group were 0.92 (95% CI: 0.87, 0.97; p < 0.001) and 0.89 (95% CI: 0.87, 0.90; p < .001), respectively [32]. A pre and post study in Bostwana examined ART initiation treatment outcomes and found a 93% retention rates among the HIV infected individuals in both groups (pre and post) [35]. A randomized controlled trial conducted in South Africa showed that multidisciplinary point-of-care testing in primary health care clinics accelerated HIV ART initiation but did not alter retention in care at six months (47 vs. 50%) (adjusted Prevalence Ratio (aPR) 0.96; 95% CI: 0.79 to 1.16) [23].

*3*.*4*.*2*.*2*. *Differentiated drug delivery models*. In Nigeria, a community pharmacy-based ART refill model was implemented where stable clients were devolved to community pharmacies for routine refills at a service fee, to promote private sector participation and sustainability of ART services. The retrospective cohort study demonstrated excellent clinical outcomes of retention rates at 98% [29]. South Africa’s differentiated community based ART delivery programme (CCMDD) for PLHIV was associated with retention in care (Risk Ratio (RR) = 1.08 p = 0.017) [25]. A randomized control trial in Uganda evaluated the impact of differentiated care model called Family Clinic Day (FCD) involving a family‐centered appointment scheduling, health education led by an expert client and patient flow where children, adolescents and their families were prioritized for care over other patients against a group receiving standard of care service as per the Uganda Ministry of Health. The analysis indicated that patients enrolled in care at an intervention facility had an 11% higher odds of being retained in care as compared to that of control facilities, though the difference was not significantly different (adjusted Odd Ratio (aOR) 1.11; 90% CI 0.63‐1.97, p = 0.75) [28].

*3*.*4*.*2*.*3*. *Point of care HIV birth testing*. A prospective cohort study of a point of care (POC) HIV birth testing in Swaziland demonstrated high retention rate of 84%, 6 months after initiating treatment [30].

#### 3.4.3. Behaviour interventions and support

*3*.*4*.*3*.*1*. *Lay counselor support*. An individualized randomized controlled trial in Kenya showed that a combination intervention of lay counselor called *Mama Mshauri* significantly lowered the attrition of mother -infant pairs at 6 months compared to the standard of care arm (18.8% vs. 28.2%, P = 0.04) [27].

*3*.*4*.*3*.*2*. *Mentor Mother programme*. A cohort study in Nigeria showed that a structured mentor mother programme was associated with higher odds of postpartum retention than routine patient support (aOR = 5.9, 95% CI: 3.0 to 11.6) [33]

*3*.*4*.*3*.*3*. *Nurse counselor care*. Kiragga, A. N., et al assessed the impact of nurse-targeted care on retention at 6 mnths among HIV positive adults in Uganda and found 62% vs 76%, p = 0.001 retention rates among those in routine care and in the nurse counselor cohort respectively [36].

*3*.*4*.*3*.*4*. *Quality improvement interventions*. Two quality improvement interventions, one in Uganda showed an increased retention rate from 29.3% to 96.7% among HIV positive adolescents [39], while the other one in Nigeria conducted through a randomized control trial did not differ across study arms in the rates of postpartum retention at 6 months (44% vs. 41% (aRR = 1.08; 95% CI:0.78 to 1.49)) [24].

#### 3.4.4. Incentives

*3*.*4*.*4*.*1*. *Financial*. One study from Tanzania sought to determine whether varying sized financial incentives for clinic attendance effected retention in care among HIV positive adults aged 18 years and above. At 6 months, 73% of participants in the control group remained in care and had viral suppression, compared with 83% in the smaller incentive group (risk difference (RD) 9·8, 95% CI 1·2 to 18·5) and 86% in the larger incentive group (RD 13·0, 4·5 to 21·5) [26]

## 4. Discussion

This paper identified 19 published studies assessing interventions to improve early retention of patients in ART programmes in SSA. The interventions were demonstrated in 11 SSA countries which included Botswana, Cameroon, Ethiopia, Kenya, Malawi, Nigeria, Uganda, South Africa, Swaziland, Tanzania and Zambia. Most of the evidence was generated through non-interventional/ observational designs. The interventions that showed promising results were grouped into the following broad areas: 1) Health system interventions involving UTT, integration of ART initiation, HTC and ANC services and reduction of ART drug costs; 2) patient centered approaches such as fast track ART initiation, Differentiated Drug Delivery models such as community based ART refill centers and point of care HIV birth testing; 3) behavioural interventions and support through lay counselors, mentor mothers and nurse counselors, application of quality improvement interventions and 4) varying sized financial incentives. Majority of the interventions targeted at improving early retention among HIV positive adults and HIV positive pregnant women. However, there is paucity of studies in SSA examining interventions focusing on improving early retention among men and key populations such as female sex workers, men having sex with men even though it is well know that such kind of skewed focus may negatively impact the adoption of major policies such as universal test-and-treat [28] and men are known to be at higher risk of attrition from ART in most settings hence need novel interventions to improve retention in HIV care [42].

Various health system interventions have been implemented with the intention of improving early retention among patients starting ART. Such inteventions include the UTT policies which aim to maximize population level treatment outcomes. The policies are implemented in many ways such as same day ART initiation in Zambia targeting incarcerated individuals which ended up being successful in retaining patients in care. However, in South Africa, same day ART among adult patients was found to increase the risk of LTFU and therefore the patients may require additional counseling and interventions to improve retention [43]. Another way in which UTT policy has been implemented is through option B+ to improve PMTCT cascade among HIV positive pregnant women. The two studies in Swaziland and Malawi which compared the CD4 guided eligibility (pre-option B+) to option B+ showed substantial increases in pregnant women retained in care through 6 months postpartum at 53% and 65% respectively [34, 41], these proportions are marginal and point to challenges along the PMTCT cascade such as refusal to start ART, continued losses after ART initiation because of women choosing to stop ART and LTFU. Such close examination of the cascade in the countries to first implement option B+ informed other countries as they implemented, transitioned to or considered implementing option B+ and should be emulated when implementing such noble interventions.

Clinical trials had demonstrated that delays of even weeks in ART initiation increased mortality among patients presenting with active opportunistic infections. Even among those without active infections, mortality among untreated persons with CD4 < 350 would approach 15/100 person‐years ‐ a large proportion of which would be avoidable in patients who presented to care. This led to the Streamlined ART Initiation Strategy (START) to initiate the greatest number of eligible patients in the shortest amount of time possible, while maintaining safety, efficacy and cost effectiveness.

In Malawi, health facilities where ANC clinics provided integrated HTC and ART initiation services observed significantly higher uptake of ART but lower retention on ART, compared with health facilities where ANC clinics only provide HTC and ART was initiated outside the ANC clinic [34]. We see that even under integration of services, initiation of ART on the same day as HIV diagnosis was associated with reduced retention in the first six months after ART initiation, independent of model of care. This shows variation across settings of the adoption and implementation of policies on integrated HIV and maternal health services as seen in a multi-country study in Malawi, Tanzania and South Africa [44].

In Cameroon, reducing the cost of ART increased enrolment of clients in the programme, but did not change retention in care. Making the clinics accessible through decentralization may have been more important than the cost of medication for retention in care [38].

The differentiated drug delivery models in South Africa, Uganda and Nigeria [25, 28, 29] have demonstrated excellent clinical outcomes of retention. The models included a community pharmacy-based ART refill, community based ART delivery programme and Family Clinic Day. Such model aim at providing quality care while reducing personal and logistical barriers to access to ART and are preferred by clients as it saves them money on transport, reduce waiting time and allow for a more thorough consultation while continuing to provide quality HIV care [45]. This review suggests that point of care HIV testing of infants at birth improve retention and viral suppression of infants in care. Birth testing that targets high-risk infants is likely to identify majority of in utero HIV transmissions and allow early ART initiation for such infants [46].

Behavioural interventions and support through lay counselors, mentor mother programmes and nurse counselors were associated with higher odds of retention compared to routine patient support. Such interventions and support play significant roles in the management of women living with HIV and their HIV-exposed infants leading to improved Maternal and Child Health outcomes in the process [47].

There have been few studies testing the use of financial incentives to improve early retention on ART even though this review demonstrate that small amounts of financial incentives improve retention and viral suppression in HIV positive individuals [26].

The main strengths of this review are the use of a comprehensive logic grid for search strategy that allowed the identification of both published and unpublished literature and a rigorous assessment of the study risk of bias and quality. Most studies documented interventions targeted at improving early retention of patients in the general HIV care programmes and in the PMTCT setting, specifically the postpartum period. The few interventional studies reviewed found an overall low risk of bias. The assessment found that the reviewed literature had some limitations. First, the quality of published studies reviewed was limited where majority of them being observational studies with some concerns as risk of bias. The selection of representative series of the non exposed cases was rarely adequately described, the comparability lacked clarity on the variables controlled for during data analysis and the outcome had no statements on adequacy in follow-up of study cohorts, making it difficult to compare outcomes. It is likely that HIV programme evaluation reports documenting interventions implemented to improve early retention of patients in ART programmes might not have been published or posted on the internet and thus might have been missed by this review. While the literature review was extensively conducted in three international databases, regional databases such as the Africa-Wide Information were not searched with the assumption that most regional studies are archived in the international databases. Similarly, the review was limited to SSA. Thus some potential interventions from north Africa and high income countries which might be applicable to SSA might have been missed by this review. This therefore limit the generalizability of the findings beyond this region and even within the SSA there is noted heterogeneity of models and approaches targeted at improving early retention of patients in ART programmes. Finaly, most of the studies documented a single intervention targeted at a single outcome in the HIV cascade of care.

## 5. Conclusion

With the introduction of universal test-and-treat and same-day initiation of ART, the findings from this review suggest that adoption of policies that expand ART uptake with the goal of reducing HIV transmission at the population level, promoting patient centered approaches such as fast track ART initiation, Differentiated Service Delivery models and providing adequate support through Mentor Mothers, lay and nurse counselors may improve early (within the first six months) retention in HIV care in SSA. However, these interventions have only been tested in few countries in the SSA region which points to how hard evidence based HIV programming is. Further implemention research, focused programme evaluations and studies with rigorous designs investigating the impact of individual and a combination of interventions to improve early retention in HIV care, including for various groups at high risk of attrition, is warranted in many of the SSA countries in order to fast track the achievement of 95-95-95 UNAIDS targets by 2030.

## Supporting information

S1 Checklist PRISMA 2020 checklist. (DOCX)
